# Mental sequelae of the COVID-19 pandemic in children with and without complex medical histories and their parents: well-being prior to the outbreak and at four time-points throughout 2020 and 2021

**DOI:** 10.1007/s00787-022-02014-6

**Published:** 2022-07-22

**Authors:** Melanie Ehrler, Cornelia F. Hagmann, Alexandra Stoeckli, Oliver Kretschmar, Markus A. Landolt, Beatrice Latal, Flavia M. Wehrle

**Affiliations:** 1 Child Development Center, University Children’s Hospital Zurich, Zurich, Switzerland; 2 Children’s Research Center, University Children’s Hospital Zurich, Zurich, Switzerland; 3 University of Zurich, Zurich, Switzerland; 4 Department of Neonatology and Intensive Care, University Children’s Hospital Zurich, Zurich, Switzerland; 5 Department of Pediatric Cardiology, University Children’s Hospital Zurich, Zurich, Switzerland; 6 Department of Psychosomatics and Psychiatry, University Children’s Hospital Zurich, Zurich, Switzerland; 7 Division of Child and Adolescent Psychology, Department of Psychology, University of Zurich, Zurich, Switzerland

**Keywords:** Well-being, Quality of life, COVID-19 pandemic, Family, Children, Parents

## Abstract

The objective of this study is to understand the long-term mental sequelae for families over the course of the COVID-19 pandemic by longitudinally investigating the well-being of children with and without complex medical histories and their parents. Well-being of 200 children (between 7 and 18 years of age; 73 typically developing, 46 born very preterm, 73 with complex congenital heart disease) and 175 of their parents was assessed prior to and during the first (April–May 2020), second (October–November 2020), third (April–May 2021), and fourth wave (October–November 2021) of the pandemic with standardized questionnaires. Linear mixed models were used to investigate longitudinal changes in child and parent well-being compared to before the pandemic. Social and COVID-19-specific determinants were investigated as predictors of impaired well-being. To illustrate clinical relevance, the proportion of children and parents scoring > 1 SD below normative mean/median was reported. Compared to before the pandemic, child proxy-reported well-being was lower during the first but not the second, third, and fourth waves. Child self-reported well-being was not lower during the pandemic compared to before. Parent well-being dropped during the first wave and remained low throughout the subsequent waves. Proxy-reported child and self-reported parent well-being was lower in families with sparse social support and poor family functioning. Parents of typically developing children reported lower well-being than parents of children born very preterm or with a complex congenital heart disease. In November 2021, 20% of children (both self- and proxy-report) and 24% of parents scored below the normal range compared to 11% (child self-report), 10% (child proxy-report), and 16% (parent self-report), respectively, before the pandemic. The pandemic continues to impact the well-being of parents of school-aged children with and without complex medical histories more than 1 year after its outbreak. Children’s well-being was specifically affected during the first wave of the pandemic and has recovered thereafter. Families with sparse social support and poor family functioning are particularly at risk for compromised well-being and support should be provided to them.

**Supplementary Information:**

The online version contains supplementary material available at 10.1007/s00787-022-02014-6.

## Introduction

More than a year into the COVID-19 pandemic, evidence for an acute negative impact on mental health has accumulated around the globe (see, e.g., [1, 2] for an overview). From the beginning of this crisis, children and adolescents were identified as being particularly at risk for impaired well-being due to the profound changes in the psychosocial environment that accompanied measures to halt the spread of the pandemic, particularly the closing of schools and the reduction of social contacts [3–7]. Indeed, numerous studies have reported reduced well-being and high rates of internalizing and externalizing problems and symptoms of anxiety and depression in children and adolescents during the first wave [8–20]. Parents were also strongly burdened by the pandemic: many of them faced increased parental responsibilities and stress working remotely while concurrently caring for their children at home [15, 19, 21–27]. Indeed, the well-being of parents was reported to be more strongly affected than that of adults without children [28, 29]. A number of factors have been associated with poor well-being during the pandemic, including social determinants such as low socio-economic status and sparse social support [1]. In addition, it is unclear whether children with pre-existing medical conditions and their parents are at particular risk for lower well-being [13, 19, 30].

To date, the majority of studies reported on the acute impact of the COVID-19 pandemic on well-being during the initial wave in early 2020. However, the long-term mental sequelae of this ongoing pandemic are less clear. Several studies have reported reduced well-being and increased behavioral problems in children throughout the first months past the initial wave [31–38]. Others have found similar levels of child well-being as prior to the outbreak [39, 40]. In parents, persistent impairments of well-being were apparent beyond the initial wave of the pandemic [35, 36, 41]. The aim of the current study was to investigate the well-being of both children and their parents as the pandemic continued to evolve. Data from two cohort studies assessed prior to the outbreak were complemented with data collected at four time-points over the course of the pandemic to investigate the immediate (first wave, April–May 2020), intermediate (second wave, October–November 2020), and long-term (third wave, April–May 2021, and fourth wave, October–November 2021) impact on children and parents and to identify factors contributing to impaired well-being.

## Methods

### Participants and study procedure

Families were recruited from two ongoing prospective cohort studies at the University Children’s Hospital Zurich, Switzerland: The EpoKids study [42] investigates the potential long-term neuroprotective effect of erythropoietin on executive functions in children born very preterm (VPT). Children born VPT were eligible for EpoKids if they had been enrolled in the trial ‘Does erythropoietin improve outcome in very preterm infants?’ (NCT00413946) at birth and had participated in the 2-year follow-up assessment [43]. They were recruited for EpoKids when they were between 7 and 12 years old. Typically developing, term-born children of the same age were recruited as siblings, friends, and through flyers at local schools and hospitals, and included into a control group [42]. The Research and Child Health Outcome (REACHOUT) study longitudinally follows children with congenital heart disease (CHD) who underwent cardiopulmonary bypass surgery at the University Children’s Hospital Zurich, Switzerland before 6 years of age between 2004 and 2009. At 10 years of age, only children without genetic or syndromal disorders were assessed. The REACHOUT study focuses on the neurodevelopmental outcome of these children [44, 45]. Families were eligible for the current study on well-being during the COVID-19 pandemic if the child had participated in the neurodevelopmental assessment and the parents had completed a set of questionnaires on child and parent well-being between January 2013 and mid-March 2020 as part of the EpoKids or the REACHOUT study (T0: prior to the implementation of measures to reduce COVID-19 in Switzerland). Parents of eligible families were invited to complete an online survey once during the first wave of the pandemic (T1: between April 17 and May 10, 2020), while lockdown measures, including school closure, were in place in Switzerland [13]. Families who had participated in the T1 assessment were approached again once during the second wave (T2: between October 30 and November 22, 2020), once during the third wave (T3: between April 23 and May 23, 2021), and once during the fourth wave (T4: between October 29 and November 21, 2021), when governmental restrictions were less severe: schools were open, but public and private assemblies were restricted. The parents could either fill out the questionnaire online or in a paper–pencil format (sent by mail with postage-paid return envelope). The vast majority of families chose the online format. Supplementary Fig. 1 details the assessment procedure for the current study. Supplementary Table 1 lists the restriction measures in Switzerland at each assessment time-point.

Families who participated in the COVID-19 survey at T1 did not differ in parental education or parent well-being prior to the pandemic (T0) from those who did not participate at T1 [13]. Furthermore, parents who participated at T2 (*P* = 0.192) and T3 (*P* = 0.671) did not differ in parent well-being at T1 from those who did not participate at T2 and T3, respectively. Parents who participated at T4 had lower parental well-being at T1 in comparison to parents who did not participate at T4 (*P* = 0.048) with small effect size (Median difference = 2.86, Cohen’s *d* = 0.287). The length of the time interval between T0 and T1 was not associated with changes in child well-being [13]. The study was approved by the local ethics committee, and all parents gave written informed consent.

### Assessment instruments

Standardized questionnaires assessing quality of life were selected from the protocols of the two cohort studies (T0), and were included in the online surveys at T1, T2, T3, and T4.

For children, the *psychological well-being* subscale of *Kidscreen-27 *[46] was used to assess well-being. Parents completed the proxy-report of the scale at all time-points. Children completed the self-report of the scale at T0, T2, T3, and T4. At T0, children completed the questionnaires during the on-site assessment for the prospective cohort studies. At T2, T3, and T4, the questionnaires were sent to the children by mail after the parents had completed the online survey. The *psychological well-being* subscale includes seven items that assess the child’s positive emotions and satisfaction and the absence of feelings of loneliness and sadness. Raw subscale scores were transformed into T values with Swiss norms (*n* = 1672, adjusted for age and sex). Low values indicate poor well-being [46].

For parents, the *mental* subscale of the *Short Form Health* questionnaire was used to assess self-reported well-being. The 36-item version (*SF-36*) was used at T0, and the 12-item short form (*SF-12*) was used at T1, T2, T3, and T4 [47]. For the analysis at T0, only the 12 items overlapping with the short form were considered. The *mental* subscale of the *SF-12* assesses four dimensions: *vitality, social function*, *role limitations due to emotional problems,* and *mental health*. Raw scores were transformed into T values based on German norms (*n* = 2524) [48]. Both the *Kidscreen-27* and the *SF-12* have acceptable to good internal consistency [46, 47].

Several predictors of child and parent well-being were assessed: maternal and paternal education were assessed as an indicator of socio-economic status. Higher scores indicate higher education (range 2 to 12). The 14-item short form of the *Social Support Questionnaire* (*F-SozU K14 *[49]) was assessed as part of both original cohort studies, and, thus, data on the perceived extent of support from the social network that is accessible if needed were available for all families at T0. Three dimensions, *emotional support*, *practical support*, and *social integration*, were summed according to the manual. Higher scores indicate more social support. The *F-SozU K14* has excellent internal consistency [49]. At T1, the quality of family functioning was assessed with the 27-item *Family Relationship Index* (*FRI *[50]). Three dimensions, *cohesion*, *expressiveness*, and *conflict*, were summed according to the manual. Higher scores indicate better quality of family functioning. The FRI has good internal consistency [50]. Previously, family functioning during the first wave of the pandemic (i.e., T1) has been reported to be impaired in these families [13], and it was, thus, investigated whether this initial impairment continued to impact child and parent well-being as the pandemic further evolved. Familial COVID-19 risk status was assessed at T1 by asking parents whether a family member was at risk for a severe disease course in case of an infection with SARS-CoV-2 due to a pre-existing health condition. A dichotomous variable differentiated families with a member at increased risk from those without.

### Statistical analysis

Parent and child characteristics are expressed as numbers and proportions of totals (dichotomous data), median and interquartile range (ordinal data), and mean and standard deviation (continuous data). Child and parent well-being of the total sample were compared to normative data (as provided by the respective manuals [46, 48]) at each time-point using one-sample *t* tests for normally distributed data and Mann–Whitney *U *tests for skewed data. Effect sizes were estimated with Cohen’s *d* (small effect > 0.2, moderate effect > 0.5, and strong effect > 0.8 [51]). The proportion of children and parents scoring below the normal range (> 1 SD below the normative mean or median) were reported separately for each time-point to illustrate clinical relevance of low well-being.

Longitudinal changes of child and parent well-being were investigated with mixed-effect models: Three models were calculated with the following outcomes: (1) proxy-reported child well-being*,* (2) self-reported child well-being*,* and (3) parent well-being. As fixed effects, the models included assessment time (categorical: T0 = prior to the pandemic; T1 = first wave, spring 2020; T2 = second wave, fall 2020; T3 = third wave, spring 2021, T4 = fourth wave, fall 2021), group (categorical: typically developing, CHD, and VPT), and age at assessment and sex of the child or the parent, respectively. T0 (factor ‘time’) and typically developing children (factor ‘group’) were defined as reference categories. As random effect, family-specific intercepts were included to take respondent-specific variability and shared variance between siblings into account (pairs of siblings: *n* = 21). Children’s individual intercepts were nested within families. The additional predictors of child and parent well-being [i.e., parental education, (T0), perceived social support (T0), family functioning (T1), and familial COVID-19 risk status (T1)] were then added as fixed factors to those models with a significant time effect.

Unstandardized regression coefficients (*B*) and generalized semi-partial *R*^2^ (*R*^2^_*B*_), to quantify effect sizes for mixed models, were reported (small: *R*^2^_*B*_ < 0.01; medium: *R*^2^_*B*_ > 0.09; large: *R*^2^_*B*_ > 0.25 [52]). The distribution of residuals was examined to evaluate normality.

All analyses were performed with *R* version 4.0.3 [53]. *P* values at a *α*-level of 0.05 were considered statistically significant. False discovery rate (FDR) control was used to correct for multiple comparison [54].

## Results

### Sample characteristics

Before the pandemic (T0), families of 346 children had participated in one of the two cohort studies and thus were eligible for the current study. Families of 200 children participated in the online survey at T1 (follow-up rate = 58%). These children were between 7 and 13 years at T0 (mean age = 10.4 ± 1.2) and between 8 and 17 years at T1 (mean age = 12.8 ± 2.0). Details are presented in Table 1. At T2, families of 138 children (follow-up rate = 70%) completed the online survey again. At each T3 and T4, families of 134 children (follow-up rate = 67%) completed the online survey again. Primarily, mothers reported on the well-being of their children (> 90%). The questionnaires of both parents were available for 27 children; therefore, only the mothers' responses were retained for further analyses. In a number of families, parents completed the survey for more than one child, resulting in 175, 122, 117, and 117 parents participating at T1, T2, T3, and T4. Sample characteristics are displayed in Table 1.

**Table 1:** Sample characteristics

	Total sample	Typically developing children	Children with congenital heart disease	Children born very preterm
Sample size children (*N*; T0&T1/T2/T3/T4)^a^	200/138/134/134	73/55/55/51	73/49/48/51	54/34/31/32
Age of child (in years, *M* [*SD*])				
T0	10.4 (1.2)	10.3 (1.7)	10.2 (0.2)	10.7 (1.2)
T1	12.8 (2.0)	11.7 (1.9)	14.1 (1.6)	12.4 (1.5)
T2	13.3 (2.0)	12.4 (1.9)	14.6 (1.5)	12.8 (1.6)
T3	13.8 (2.0)	12.7 (2.0)	15.1 (1.6)	13.5 (1.5)
T4	14.4 (2.0)	13.3 (2.0)	15.7 (1.6)	13.9 (1.5)
Sex child (no. female (%))				
T0 & T1	96 (48%)	43 (59%)	28 (38%)	25 (46%)
T2	69 (50%)	33 (60%)	20 (40%)	16 (47%)
T3	62 (46%)	33 (60%)	16 (33%)	13 (42%)
T4	66 (49%)	31 (61%)	20 (39%)	15 (47%)
Sample size parents (*N*; T0&T1/T2/T3/T4)	175/122/117/117	54/41/41/39	73/49/48/51	48/32/28/27
Age of responding parent				
(in years, *M* [*SD*]) T0	42.7 (5.2)	41.7 (5.2)	42.2 (4.6)	44.7 (5.4)
T1	45.1 (5.4)	43.3 (5.2)	46.1 (5.0)	46.4 (5.5)
T2	45.8 (5.3)	44.4 (5.4)	47.4 (4.8)	45.9 (5.3)
T3	46.2 (5.3)	45.0 (5.5)	48.2 (5.1)	45.5 (4.6)
T4	47.1 (5.5)	45.2 (5.6)	48.8 (4.8)	47.5 (5.6)
Sex of responding parent				
(no. female (%)). T0 & T1	162 (93%)	52 (96%)	69 (95%)	41 (85%)
T2	116 (95%)	39 (95%)	48 (96%)	29 (91%)
T3	109 (93%)	39 (95%)	44 (92%)	26 (93%)
T4	109 (93%)	36 (92%)	48 (94%)	25 (93%)
Parental education (*M* [*SD*])^b^	8.8 (2.0)	9.6 (2.1)	8.7 (1.9)	8.0 (1.7)
Time length between T0 and T1 (in years, *Mdn* [*IQR*])	1.8 (1.1–3.0)	1.0 (0.8–1.9)	4.1 (2.4–5.2)	1.6 (1.3–2.1)

T0 = before the COVID-19 pandemic (2013–2020), T1 = first wave of the COVID-19 pandemic (April–May 2020), T2 = second wave of the COVID-19 pandemic (October–November 2020), T3 = third wave of the COVID-19 pandemic (April–May 2021), and T4 = fourth wave of the COVID-19 pandemic (October–November 2021). *M* = Mean, *SD* = Standard deviation, *Mdn* = Median, *IQR* = Interquartile range. Age range: T0 = 7 to 13 years, T1 = 8 to 17 years. ^a^child self-report of well-being was completed by 172 children (T0), 79 children (T2), 80 children (T3), and 60 children (T4). Self-report was not assessed at T1. ^b^assessed at T0, parental education combines maternal and paternal education (range: 2–12), and higher value indicates higher education. Data on parental education were missing for 4% of all participants

Of the children with CHD, 21% had a univentricular heart defect. Children born VPT were born at a mean gestational age of 28.9 weeks (SD = 1.6). In 36 families, at least one family member had been infected with SARS-CoV-2 until November 2021. In 75 families, at least one family member was reported to be at risk for a severe course of disease in case of a SARS-CoV-2 infection (assessed at T1).

### Well-being of children and their parents before and during the COVID-19 pandemic

Table 2 displays the comparison to normative data as provided by the respective manuals for child and parent psychological well-being.

**Table 2:** Well-being of the total sample compared to normative data as provided by the respective manuals

Outcome	Time	*N*	*Mean* *Median*	*SD* *IQR*	Uncorrected *P*	FDR-corrected *P*	Cohen’s *d*	% Below norm^e^
Child proxy-reported well-being^a^	T0	195	*M* = 50.57	*SD* = 10.67	0.458	0.458	0.05	11%
	T1	198	*M* = 45.57	*SD* = 11.18	**< 0.001**	**< 0.001**	0.40	30%
	T2	137	*M* = 49.25	*SD* = 10.31	0.395	0.458	0.07	18%
	T3	134	*M* = 47.75	*SD* = 12.38	**0.037**	0.092	0.18	25%
	T4	134	*M* = 48.85	SD = 10.19	0.196	0.327	0.11	20%
Child self-reported well-being^a,b^	T0	172	*M* = 51.79	SD = 9.77	**0.017**	0.068	0.18	11%
	T1	–	–	–	–	–	–	–
	T2	79	*M* = 48.58	*SD* = 8.64	0.147	0.294	0.16	14%
	T3	80	*M* = 49.75	*SD* = 9.37	0.813	0.813	0.03	18%
	T4	60	*M* = 49.27	SD = 9.50	0.556	0.741	0.08	20%
Parent self-reported well-being^c^	T0	175	*Mdn* = 52.48	*IQR* = 48.36–55.18	**0.003**^**d**^	**0.003**^**d**^	0.34	16%
	T1	175	*Mdn* = 48.93	*IQR* = 42.56–52.98	**< 0.001**^**d**^	**< 0.001**^**d**^	0.73	33%
	T2	122	*Mdn* = 50.28	*IQR* = 44.09–52.98	**< 0.001**^**d**^	**< 0.001**^**d**^	0.62	24%
	T3	117	*Mdn* = 49.87	*IQR* = 43.75–54.32	**< 0.001**^**d**^	**< 0.001**^**d**^	0.61	27%
	T4	117	*Mdn* = 49.87	*IQR* = 44.90–54.32	**< 0.001**^**d**^	**< 0.001**^**d**^	0.63	24%

Child well-being was assessed with the psychological well-being scale of the Kidscreen-27 (self- and proxy-report) [46]. Parent well-being was assessed with the mental scale of the SF-12 [48]. T0=before the COVID-19 pandemic (2013–2020, T1=first wave of the COVID-19 pandemic (April–May 2020), T2=second wave of the COVID-19 pandemic (October–November 2020), T3=third wave of the COVID-19 pandemic (April–May 2021), and T4=fourth wave of the COVID-19 pandemic (October–November 2021). Statistically significant results are displayed in bold. *M*=mean. *Mdn*=median. *SD*=standard deviation. *IQR*=interquartile range

^a^Data were normally distributed, and thus, *t *test was used. Normative mean is 50 [46], which was used as parameter for testing the null hypothesis

^b^Child self-reported well-being was not assessed at T1

^c^Data were non-normally distributed, and thus, nonparametric Mann–Whitney *U*-test was used. Normative median is 53, which was used as parameters for testing the null hypotheses

^d^Test statistics refer to pseudomedian corrected for one-sample cases. For well-being: T0 pseudo *Mdn*=51.85, T1 pseudo *Mdn*=47.69, T2 pseudo *Mdn*=48.97, T3 pseudo *Mdn*=48.77, T4 pseudo *Mdn*=48.97

^e^Proportion of individuals scoring below the normal range (>1SD below normative mean or median)

**Fig. 1:**
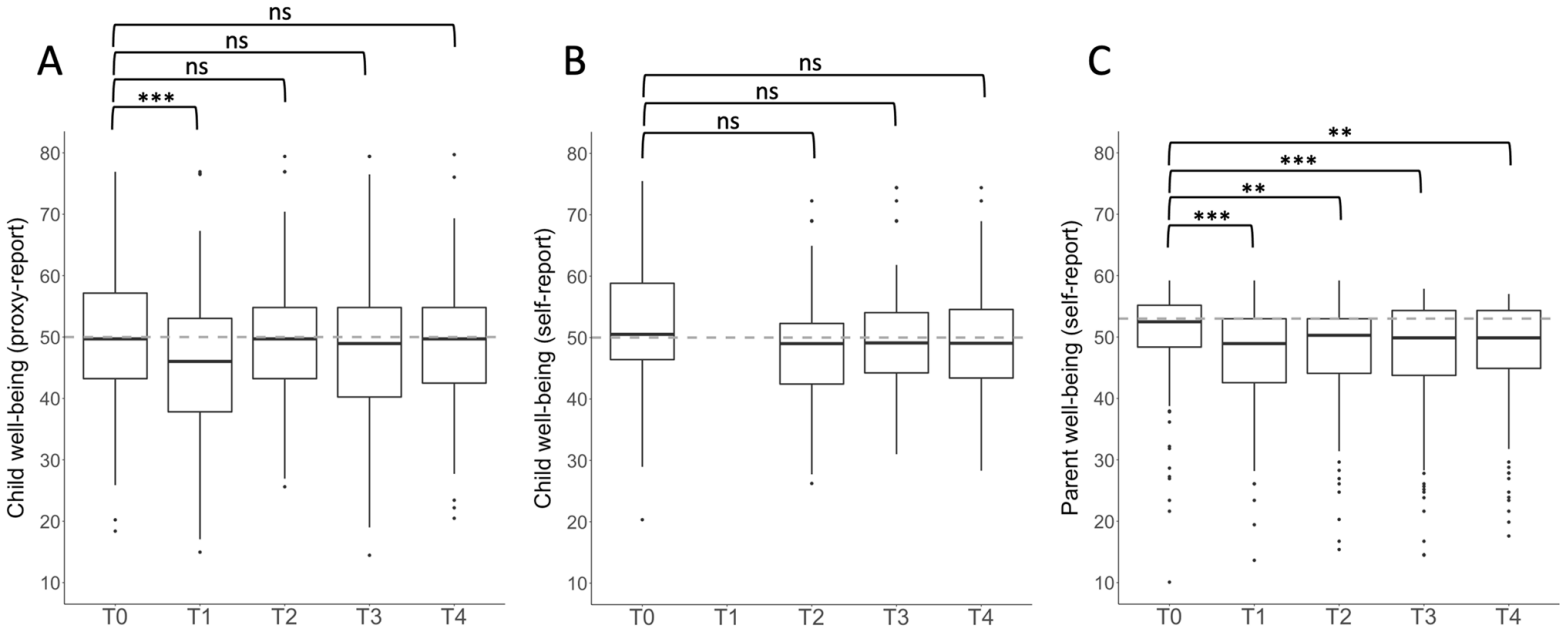
Child proxy (**A**) and self-reported (**B**), and parent (**C**) well-being before and during the COVID-19 pandemic. The box represents the interquartile range. The thick line within the box corresponds to the sample’s median. The gray dashed line represents the normative median (child well-being: *Mdn* = 50 [46]; parent well-being: *Mdn* = 53 [48]). Dots represent outliers. T0 = before the COVID-19 pandemic (2013–2020), T1 = first wave of the COVID-19 pandemic (April–May 2020), T2 = second wave of the COVID-19 pandemic (October–November 2020), T3 = third wave of the COVID-19 pandemic (April–May 2021), and T4 = fourth wave of the COVID-19 pandemic (October–November 2021). Child self-reported well-being was not assessed at T1. Well-being is expressed as *T* values. ns = not significant, **P < 0.01, ***P < 0.001 (FDR-corrected *P* values)

Table 3 reports the statistical estimates of the three linear mixed models assessing longitudinal changes in child and parent well-being. Compared to before the pandemic, child proxy-reported well-being was significantly lower during the first but not the second, the third, and the fourth wave of the pandemic (Fig. 1A). The model’s effect size was small [*R*^2^_*B*_(CI-95) = 0.044 (0.085 to 0.028)]. The effect size in the model with the additional predictors increased to moderate [*R*^2^_*B*_ (CI-95) = 0.132 (0.187 to 0.102)]. Well-being was independent of sex and age of the child. Proxy-reported well-being of children born VPT or with CHD did not differ from typically developing children after FDR correction. Sparse social support before the pandemic and poor family functioning during the first wave significantly predicted lower well-being. There was no significant interaction either between assessment time and perceived social support (*P* = 0.087) or assessment time and family functioning (*P* = 0.088) on well-being.

**Table 3:** Statistical estimates of fixed effects derived from linear mixed models investigating well-being over the course of the COVID-19 pandemic in comparison to before the pandemic

Variables	*B*	*CI-95*	Uncorrected *P*	FDR corrected *P*	*R*^*2*^_*B*_^e^	*B*	*CI-95*	Uncorrected *P*	FDR corrected *P*	*R*^*2*^_*B*_^*e*^
	Simple model					Model with additional predictors				
**Child proxy-reported well-being**										
Time^a^										
T1	− 4.91	− 6.96,− 2.87	**< 0.001**	**< 0.001**	0.025	− 4.90	− 7.21,− 2.58	**< 0.001**	**< 0.001**	0.021
T2	− 1.38	− 3.78,1.01	0.260	0.434	0.002	− 1.60	− 4.27,1.08	0.246	0.424	0.002
T3	− 2.83	− 5.40,− 0.26	**0.032**	0.128	0.006	− 3.21	− 6.12,− 0.31	**0.032**	0.077	0.006
T4	− 1.28	− 4.07,1.51	0.371	0.495	0.001	− 1.74	− 4.90,1.41	0.283	0.424	0.002
Age	0.00	− 0.53,0.54	0.994	0.994	0.000	0.05	− 0.55,0.65	0.871	0.871	0.000
Sex	0.29	− 2.03,2.61	0.798	0.912	0.000	1.11	− 1.36,3.57	0.381	0.457	0.003
Group^b^										
VPT born	2.45	− 0.69,5.59	0.128	0.341	0.009	3.71	0.45,6.96	**0.027**	0.077	0.016
CHD	1.75	− 1.36,4.86	0.271	0.434	0.005	1.19	− 1.99,4.38	0.466	0.508	0.002
Parental education (T0)						0.30	0.36,0.97	0.370	0.457	0.003
Perceived social support (T0)						0.08	0.04,0.13	** < 0.001**	**0.004**	0.037
Family functioning (T1)						0.50	0.17,0.82	**0.003**	**0.012**	0.030
COVID risk status (T1						1.51	1.04,4.05	0.248	0.424	0.005
**Child self-reported well-being**^c^										
Time^a,d^										
T1	–	–	–		–					
T2	− 2.62	− 5.50,0.27	0.078	0.273	0.004					
T3	− 1.43	− 4.51,1.64	0.364	0.425	0.001					
T4	− 2.18	− 5.66,1.31	0.225	0.394	0.002					
Age	− 0.13	− 0.74,0.48	0.677	0.677	0.000					
Sex	− 1.48	− 3.55,0.59	0.164	0.383	0.003					
Group^b^										
VPT born	2.79	0.09,5.50	**0.045**	0.273	0.006					
CHD	1.29	− 1.17,3.76	0.307	0.425	0.002					
**Parent well-being**										
Time^a^										
T1	− 5.07	− 3.55,− 2.03	**< 0.001**	**< 0.001**	0.016	− 3.30	− 3.30,− 1.71	**< 0.001**	**0.001**	0.013
T2	− 4.21	− 2.48,− 0.76	**0.005**	**0.010**	0.007	− 2.43	− 2.43,− 0.67	**0.008**	**0.011**	0.006
T3	− 5.20	− 3.41,− 1.62	**< 0.001**	**0.001**	0.012	− 3.36	− 3.36,− 1.53	**< 0.001**	**0.002**	0.011
T4	− 4.88	− 3.04,− 1.20	**0.001**	**0.003**	0.009	− 3.13	− 3.13,− 1.25	**0.001**	**0.002**	0.009
Age	− 0.24	− 0.05,0.15	0.640	0.640	0.001	0.00	0.00,0.20	1.000	1.000	0.000
Sex	− 9.88	5.17,− 0.46	**0.033**	**0.044**	0.01	− 6.53	− 6.53,− 1.98	**0.006**	**0.009**	0.020
Group^b^										
VPT born	0.76	3.40,6.05	**0.012**	**0.019**	0.018	4.17	4.17,6.96	**0.004**	**0.007**	0.022
CHD	0.14	2.60,5.05	**0.040**	**0.046**	0.013	3.81	3.81,6.35	**0.004**	**0.007**	0.023
Parental education at T0						0.02	0.02,0.58	0.952	1.000	0.000
Perceived social support at T0						0.07	0.07,0.11	**0.001**	**0.002**	0.029
Family functioning at T1						0.45	0.45,0.71	**0.001**	**0.002**	0.029
COVID risk status at T1						− 1.02	− 1.02,1.10	0.350	0.420	0.002

Additional predictors were: parental education (at T0), perceived social support (at T0), family functioning (at T1), and COVID-19 risk status of the family (at T1). T0 = before the COVID-19 pandemic, T1 = first wave of the COVID-19 pandemic (April–May 2020), T2 = second wave of the COVID-19 pandemic (October–November 2020), T3 = third wave of the COVID-19 pandemic (April–May 2021), and T4 = fourth wave of the COVID-19 pandemic (October–November 2021). Statistically significant results are displayed in bold. *B* = unstandardized estimate. *CI-95* = 95% confidence interval. *P* = *P*-value. VPT = very preterm born. CHD = congenital heart disease. 80 of 175 parents participated at all five measurement time-points (46%), 43 parents participated at four of five measurement time-points (25%), 26 parents participated at three of five measurement time-points (15%), and 26 parents participated at two of five measurement time-points (15%)

^a^Reference = T0

^b^Reference = typically developing children

^c^Due to an insignificant time effect, the model including predictors was not run

^d^Child self-reported well-being was not assessed at T1

^e^effect size: small < 0.01, medium > 0.09, large > 0.25 [52]

For child self-reported well-being, there was no significant change in well-being across time with small effect size [*R*^2^_*B*_ (CI-95) = 0.022 (0.055 to 0.012, Fig. 1B)]. Self-reported well-being of children born VPT or with CHD did not differ from typically developing children after FDR correction. Self-reported well-being and proxy-reported well-being correlated weakly to moderately at T0 (Spearman’s *r* = 0.15, *P* = 0.050), T2 (Spearman’s *r* = 0.11, *P* = 0.343), T3 (Spearman’s *r* = 0.35, *P* = 0.001), and T4 (Spearman’s *r* = 0.49, *P* = 0.001).

Parent well-being dropped significantly during the first wave compared to before the pandemic, and remained significantly lower during the second, third, and fourth waves (Fig. 1C). The model’s effect size was small [*R*^2^_*B*_(CI-95) = 0.057 (0.095 to 0.038)]. Adding the predictors increased the model’s effect size to moderate [*R*^2^_*B*_(CI-95) = 0.140 (0.189 to 0.112)]. The well-being of parents of children born either VPT or with a CHD was higher than the well-being of parents of typically developing children. Well-being was independent of parent age. Female sex, sparse social support before the pandemic, and poor family functioning during the first wave significantly predicted lower well-being. There was no significant interaction between assessment time and group (*P* = 0.065), assessment time and sex (*P* = 0.094), assessment time and social support (*P* = 0.276), or assessment time and family functioning (*P* = 0.298) on well-being.

Parents’ self-reported well-being and parents’ proxy-report well-being of their children correlated weakly to moderately at T0 (Spearman’s *r* = 0.28, *P* < 0.001), T1 (Spearman’s *r* = 0.20, *P* = 0.008), T2 (Spearman’s *r* = 0.21, *P* = 0.020), T3 (Spearman’s *r* = 0.30, *P* < 0.001), and T4 (Spearman’s *r* = 0.17, *P* = 0.077).

## Discussion

This study is the first to investigate the mental sequelae for families more than 1 year into the COVID-19 pandemic. The findings provide longitudinal evidence that the well-being of parents of school-aged children with and without complex medical histories has been compromised over the first 18 months since its outbreak. Furthermore, parents reported lower well-being in their children during the first wave of the pandemic but not at later time-points throughout 2020 and 2021. Children and adolescents themselves reported similar levels of well-being after the initial wave of the pandemic compared to before (self-reported child well-being was not assessed during the first wave). Families with sparse social support and poor family functioning are particularly at risk for poor well-being.

Concerns about the mental sequelae of the pandemic for families have been raised, since measures were initially implemented to reduce the spread of COVID-19 in spring 2020 [3–7, 55]. The current study is the first to link child and parent well-being before the outbreak of the pandemic to the well-being of the same individuals at four time-points throughout 2020 and 2021. Tracking these families provided clear evidence for compromised well-being in parents throughout the pandemic: parents reported lower well-being at each of the four waves throughout 2020 and 2021 compared to before the outbreak of the pandemic, with one in four parent reporting clinically relevant low well-being in late 2021 (i.e., < 1 SD below the normative data provided by the respective manual). This is in line with reports of high levels of parental stress [41] and mental health symptoms [35, 36] in parents beyond the initial wave. In fact, the well-being of parents has been reported to be more strongly affected by the COVID-19 pandemic compared to adults without children (e.g., [28, 29]).

To comprehensively evaluate child well-being throughout the pandemic, self- and parent-reports were considered in the current study. The findings are somewhat more complex: parents reported that compared to before the pandemic, the well-being of their children was substantially lower during the first wave and to a lesser degree during the third wave of the pandemic (this time-point was, however, no longer significantly lower after correcting for multiple comparison). In contrast, parent-reported child well-being was not reduced during the second and the fourth waves. These findings confirm the drop in child well-being during the first wave of the pandemic reported by numerous previous studies [8–20]. However, the findings of the current study are not in line with some studies that have reported parent-reported mental health impairments in children beyond the first wave [35, 36]. Children themselves reported similar levels of well-being at the second, third, and fourth wave of the pandemic compared to before its outbreak. Unfortunately, the current study did not assess self-reported child well-being during the initial wave of the pandemic in April/May 2020. Thus, the findings cannot be directly compared to the large body of evidence suggesting a substantial drop in self-reported child well-being during the initial wave [11, 12, 16–18]. However, they are in line with some studies that reported no or negligible reductions of self-reported well-being during the first months past the initial wave of the pandemic [39, 40], even though other studies found persistent reductions [11, 31, 32, 34, 36, 38]. Importantly, although self-reported well-being of children was preserved at group-level, as many as one in five children reported clinically relevant low well-being in November 2021 (before the pandemic: 1 in 10 children). Similarly, previous studies have found no change in well-being at group-level but reported considerable variability between individuals alongside a fair number of children with substantially increased mental health symptoms [56, 57].

Taken together, the findings of the current study provide evidence that the well-being of parents was compromised throughout the first 18 months of the pandemic, while the well-being of children was specifically affected during the first wave in April/May 2020, as reported by their parents, and has subsequently recovered. In Switzerland, schools were closed during the first wave of the pandemic, when mandatory home-schooling was implemented, but remained open during all the following waves. In contrast, social distancing and home-office orders were reinforced as the second wave surged and (at least partly) remained in place throughout the subsequent waves. This likely strained parents and continued to compromise their well-being, while the less-restrictive measures for children, including open schools, may have beneficially impacted child well-being.

Interestingly, the current study found that parents of children with complex medical histories experienced a higher level of well-being than parents of typically developing children. This was true both before and during the pandemic. Previously, it was suggested that clinical populations may experience above-norm well-being, because they have adapted their internal standards and values regarding well-being. This resulted in a change in the perception of their own well-being—a response shift [58, 59]. Importantly, the current study found that parents of children with and without complex medical histories experienced a similar drop in well-being throughout the COVID-19 pandemic. Thus, their well-being appears to be affected by this crisis in a similar manner. Furthermore, families with a member who was at increased risk for a severe disease course in case of infection with SARS-CoV-2 were affected similarly to families without in their well-being during the pandemic. The familial COVID-19 risk status was assessed during the first wave of the pandemic. It was expected that those at higher risk perceived the pandemic as more serious and were, thus, more strongly impacted in their well-being. However, the current findings do not support this. This is in line with previous studies reporting similar levels of well-being in the early phase of the pandemic in individuals at high risk for a severe disease course compared to individuals at low risk (e.g., young adults with CHD or older adults [60, 61]). Importantly, individuals with pre-existing medical conditions have, despite retaining their levels of well-being, previously been shown to experience specific concerns related to the ongoing pandemic, including increased fear of contracting the virus, and may thus require specific attention from their health care providers [60, 62].

The current findings imply that the well-being of mothers may be particularly affected throughout the pandemic. This is in line with previous studies reporting that mothers experienced higher levels of depressive and anxiety symptoms during the pandemic compared to fathers, and compared to women without children [10, 63, 64]. Families with sparse social support before the outbreak of the pandemic and poor family functioning during the first wave of the pandemic were found to be at particular risk of poor well-being. Social factors have previously been identified as contributors to poor well-being during the pandemic [1]. However, these factors are likely not unique to the COVID-19 pandemic, because they have been shown to contribute to poor well-being of children and parents in general (e.g., [65, 66]). Even so, the confirmation of social risk factors for poor well-being during the ongoing pandemic is important for identifying families who are at particular risk for long-term mental sequelae. Moreover, social support may be provided not only by the individuals’ social network but also by professionals, including social workers. Thus, strengthening services during and in the aftermath of the current pandemic may prevent long-term mental health sequelae for those at risk.

Future research investigating subgroups of individuals with different trajectories of well-being over the course of the pandemic and identifying potential additional predictors of these trajectories will significantly advance the understanding of the long-term mental sequelae of this crisis. This should include investigating the positive effects of the pandemic reported by many studies, such as increased family time and reduced stress from fewer obligations related to school or other activities [e.g., [67]]. In fact, a cross-sectional study during lockdown has shown that children and adolescents who reported positive effects during the lockdown, including improved relationships with family and friends, reduced bullying, and more sleep and exercise, experienced improvements in mental well-being compared to before the pandemic [68]. Future studies should continue to investigate the potential protective factors of both parent and child well-being.

### Limitations

The current longitudinal investigation draws on two cohort studies not originally designed to investigate the mental sequelae of COVID-19 for families. Thus, some limitations require consideration: the questionnaires for the assessments during the pandemic were selected from the study protocol of the cohort studies to allow changes to be investigated. These questionnaires assess well-being rather than mental health symptoms; thus, the conclusions that can be drawn about the prevalence of mental health problems during the pandemic are limited. The study sample includes children with and without complex medical histories and is not representative of the general population of children and parents in Switzerland. However, the longitudinal findings presented here are in line with and complement findings from cross-sectional studies conducted during the initial wave of the pandemic with nationally representative samples that report child and parent well-being compromised in comparison to normative data [11, 25].

The sample size of the current study was relatively small compared to previous cross-sectional studies (e.g., [15, 16, 20]). However, its longitudinal design ensured well-powered analyses owing to within-subject correlations [69]. Of the participating families, 71% participated at four or five measurement time-points. Well-being at T1 was not different between those parents who participated at T2 and T3 compared to those who did not participate, but was lower in those who participated at T4 compared to those who did not participate (small effect size; *P* = 0.048, Cohen’s *d* = 0.287).

Participating families come from high and rather homogenous socio-economic backgrounds, as is often seen in prospective cohort studies [70]. This may explain the absence of any effect of parental education on well-being, which had been expected from previous findings [1].

Finally, the participation rate in child self-reports was lower compared to the parent-reports. Also, no self-report of child well-being was assessed during the first wave of the pandemic. Thus, no conclusion can be drawn from this study about the potential immediate effects on child self-reported well-being. Correlations between parent- and self-reports of child well-being were weak to moderate. This is in line with other studies reporting modest correlations, likely because parent and self-reports reflect different realities of perceived well-being [71, 72]. Therefore, studying both, child and parental perspectives, is crucial to comprehensively understand child well-being [73]. Importantly, however, parents’ self-reports of their well-being and parents’ proxy-reports of their children’s well-being also correlated only weakly to moderately. This suggests that parents are able to distinguish the perception of their own well-being from that of their children rather than the low parental well-being negatively impacting their report of child well-being.

### Conclusions

This study provides evidence of the long-term mental sequelae of the COVID-19 pandemic for parents of school-aged children with and without complex medical histories. One in four parent reports substantially compromised well-being in late 2021—more than 1 year into the crisis. Children’s well-being was specifically affected during the initial wave of the pandemic, as reported by their parents, and subsequently recovered. Importantly, even small psychological impacts of the pandemic have been argued to require careful attention as they may pose a substantial public health problem if reproduced across the whole population [2]. Consequently, it is crucial to provide psychological support to those in need alongside the comprehensive economic measures that have been implemented by governments to recover from the ongoing COVID-19 pandemic.

### Lessons learned and consequences for the future

The findings of the current study provide evidence that parents of school-aged children require attention throughout a pandemic as their psychological well-being remains compromised well beyond the initial wave. This is specifically true for those with limited social support and poor family functioning. Strategies to support vulnerable families during times of crises need to be developed and implemented, and most importantly, they must remain in place even if social distancing measures are necessary to reduce infection rates (e.g., virtual counselling). Further, children’s well-being may be particularly compromised during times of school closures. Keeping schools open whenever possible, or re-opening them early after periods of strict lock-down should, thus, be part of future pandemic strategies to support psychological well-being of children throughout a pandemic.

## Supplementary Information

Below is the link to the electronic supplementary material.

Supplementary file1 (PDF 467 KB)

## Data Availability

For re-analyses of the data set (for different purposes), additional ethical approval (on an individual user and purpose basis) will be required. The authors are happy to support additional ethical approval applications from researchers for access to this data set.

## Code availability

R Code is available from the corresponding author upon reasonable request.
