# Supplementary material for: Mental sequelae of the COVID-19 pandemic in children with and without complex medical histories and their parents: well-being prior to the outbreak and at four time-points throughout 2020 and 2021
Source: Eur Child Adolesc Psychiatry. 2022 Jul 22;32(6):1037–49. doi: 10.1007/s00787-022-02014-6 (PMC9305026; doi:10.1007/s00787-022-02014-6)
Supplement: Supplementary file 1 — Supplementary file1 (PDF 467 KB) [file 787_2022_2014_MOESM1_ESM.pdf]

Supplementary information

Title: Mental sequelae of the Covid-19 pandemic in children with and without complex medical histories and their parents: Well-being prior to the outbreak and throughout 2020 and 2021

Journal: European Child & Adolescet Psychiatry

Authors: Melanie Ehrler, Cornelia F. Hagmann, Alexandra Stoeckli, Oliver Kretschmar, Markus A. Landolt, Beatrice Latal, Flavia M. Wehrle

Address correspondence to Flavia M. Wehrle, Child Development Center, University Children's Hospital Zurich, 75 Steinwiesstrasse, 8032 Zurich, Switzerland, [flavia.wehrle@kispi.uzh.ch].

Supplementary Figure 1: Flow chart

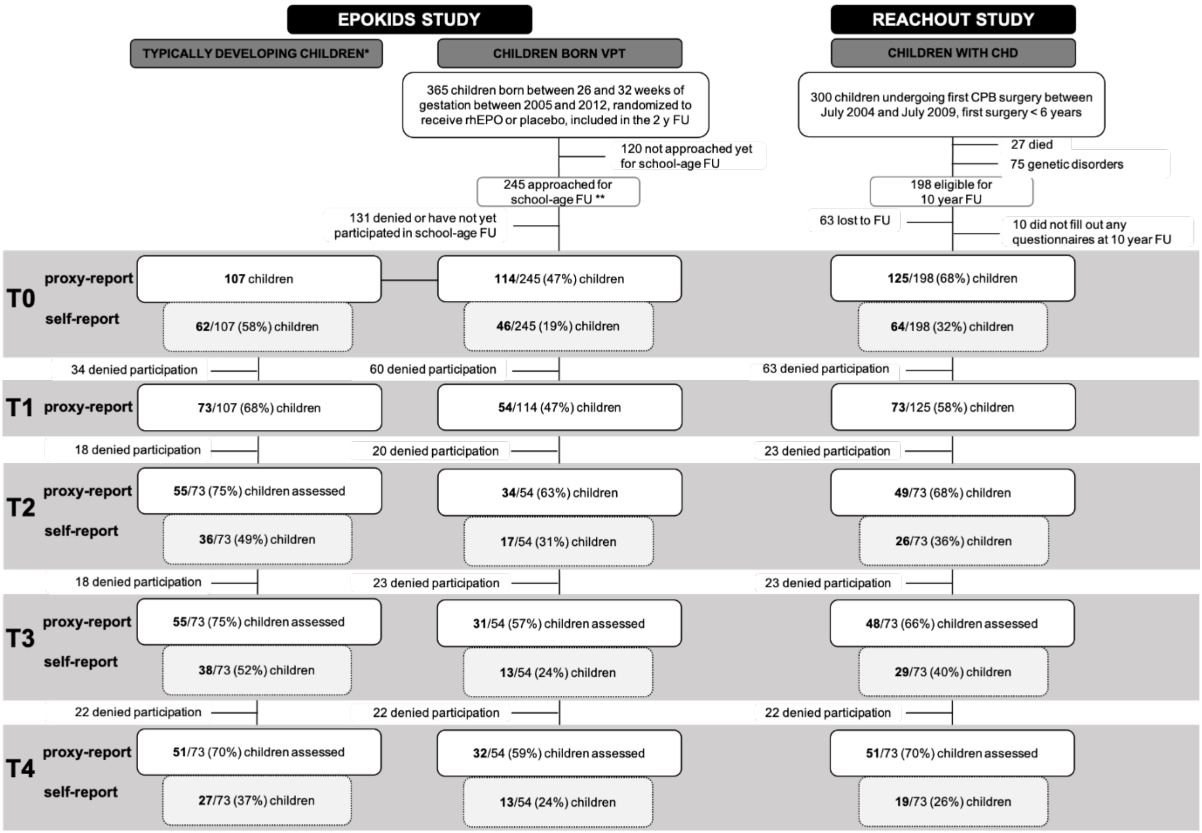

Note: Flow chart refers to the child self- and proxy-reports. Numbers of participating parents is lower because some parents reported on more than one child (e.g., T1: 25 parents reported on more than one child, thus, self-reports of 175 parents of 200 children are available). T0 = before the COVID-19 pandemic, T1 = first wave of the COVID-19 pandemic (April–May 2020), T2 = second wave of the COVID-19 pandemic (October–November 2020), T3 = third wave of the COVID-19 pandemic (April–May 2021), T4 = fourth wave of the COVID-19 pandemic (October–November 2021).* Typically developing children were recruited as control group for the EpoKids study at school-age **Ongoing study. FU = follow up, CHD=congenital heart disease, CPB = cardiopulmonary bypass surgery. VPT: very preterm. Epokids study[1]. Reachout study[2].

References

1) Wehrle FM, Held U, O'Gorman RT, et al. Long-term neuroprotective effect of erythropoietin on executive functions in very preterm children (EpoKids): protocol of a prospective follow-up study. *BMJ open.* 2018;8(4):e022157-e022157.

2) Werninger I, Ehrler M, Wehrle FM, et al. Social and behavioral difficulties in 10-year-old children with congenital heart disease: Prevalence and risk factors. *Frontiers in pediatrics.* 2020;8.

Supplementary Table 1: Overview of nationwide measures to reduce the spread of COVID-19 in Switzerland.

Wave	Nationwide restrictions
1st wave<br>(April/May 2020)	School closure<br>Prohibition of public events<br>Prohibition of private events<br>Closure of nonessential and retail services<br>Reduction of public transport service
2nd wave<br>(October/November 2020)	School closure for adult education only<br>Mandatory face masks in public space for people at the age of 12 years or older<br>Prohibition of public events with >50 people<br>Prohibition of private events with >10 people<br>Prohibition of leisure activities with >15 people<br>Restriction in restaurants to 4 people per table and earlier closing times
3rd wave<br>(April/May 2021)	Prohibition of adult education with > 50 people<br>Mandatory face masks in public space for people at the age of 12 years or older<br>Prohibition of public events with >50 people (inside) and >100 people (outside)<br>Prohibition of private events with >15 people<br>Prohibition of leisure activities with >15 people

	Inner area of restaurants closed; outdoor area restricted to 4 people per table
4th wave<br>(October/November 2021)	Adult education with COVID certificate<br>Mandatory face masks in public indoor space for people at the age of 12 years or older (no mandatory face masks when COVID certificate is required)<br>COVID certificate for all public events inside (outside only with > 500 people)<br>Private events restricted to <30 people inside (outside <50 people)<br>No restrictions for leisure activities with <30 people. COVID certificate for leisure activities with >30 people.<br>Inner area of restaurants, bars and clubs is open for people with COVID certificate

Note. Some cantons may have ordered stricter measures. Source: Federal Office of Public Health (FOPH) Switzerland https://www.bag.admin.ch/bag/en/home/krankheiten/ausbrueche-epidemien-pandemien/aktuelle-ausbrueche-epidemien/novel-cov/massnahmen-des-bundes.html (accessed January 14th 2022). COVID certificate was issued if subject was fully vaccinated, recovered from COVID-19 within the past 6 months, was tested negative for COVID-19 within the past three days (PCR) or one day (rapid test).
